# An evaluation of the notifiable disease surveillance system in Chegutu District, Zimbabwe, 2020: a cross-sectional study

**DOI:** 10.11604/pamj.2022.41.215.33712

**Published:** 2022-03-16

**Authors:** Memory Chimsimbe, Pride Mucheto, Emmanuel Govha, Addmore Chadambuka, Mujinga Karakadzai, Tsitsi Patience Juru, Notion Tafara Gombe, Mufuta Tshimanga

**Affiliations:** 1 University of Zimbabwe, Department of Primary Health Care Sciences, Global and Public Health, Harare, Zimbabwe,; 2 Zimbabwe Department of Oral Health, University of Zimbabwe, Harare, Zimbabwe,; 3 Zimbabwe Community Health Intervention Research, Harare, Zimbabwe,; 4 African Field Epidemiology Network, Harare, Zimbabwe

**Keywords:** Surveillance, notifiable disease, system attribute, Zimbabwe

## Abstract

**Introduction:**

in 2018-2019 Chegutu District had one notification form Tally 1 (T1) that was completed instead of seven for detected notifiable diseases. Different figures of cholera were reported through weekly rapid disease notification system with 106 patients and Notifiable Diseases Surveillance System (NDSS) with 111 patients, causing data discrepancy. We evaluated the NDSS to determine reasons for underperformance and data discrepancy.

**Methods:**

we conducted descriptive cross-sectional study using updated centres for disease control and prevention guidelines for surveillance system evaluation. We recruited forty-six health workers. Interviewer-administered questionnaires and checklists were used to collect data on reasons for underperformance, reasons for data discrepancy, knowledge of NDSS, surveillance system attributes and usefulness. Epi InfoTM7 generated frequencies, proportions, and means. Likert scale was used to assess health worker knowledge.

**Results:**

of the forty-six health workers, 34 (78%) had fair knowledge of NDSS. The reason for system underperformance was lack of training in NDSS 42 (91%). Data discrepancy was attributed to typographical mistakes made during data entry on WhatsApp platform 32 (70%). Eighty per cent (37) were willing to complete T1 forms. Six participants who were timed took ten minutes to complete T1 forms. Among 17 health facilities, only three had fifteen T1 forms that were adequate to notify first five cases in an outbreak. Notifiable diseases surveillance system data was used for planning health education 28 (68%).

**Conclusion:**

the NDSS was unstable due to health workers' inadequate knowledge and unavailability of T1 forms. Notifiable diseases surveillance system was found to be simple, acceptable, and useful. We recommended NDSS training of health workers.

## Introduction

Public health surveillance relates to the ongoing, systematic collection, analysis, interpretation, and dissemination of information regarding a health-related event for use in public health to reduce morbidity, mortality and improve health [1]. One such public health surveillance is the Notifiable Disease Surveillance System (NDSS) which aims to identify notifiable diseases early before they become numerous to institute prevention and control measures [2]. In Zimbabwe, the Public Health Act established the NDSS in the 1950s [2]. The Public Health Act (15.09) outlines a list of 19 notifiable diseases, with a provision for the addition of emerging diseases by the Minister of Health and Child Care [3]. The objective of the NDSS is timely preparedness for epidemics and the effective planning, implementation and evaluation of epidemic disease control programs [4]. The NDSS links the health information system from the health facility to the national level. The NDSS reporting system was strengthened in 1991 by the Weekly Rapid Disease Notification System (WRDNS) which provides an early warning system of potential threats to humans health through monitoring of notifiable diseases, other diseases and conditions under surveillance weekly [5].

**Figure 1:**
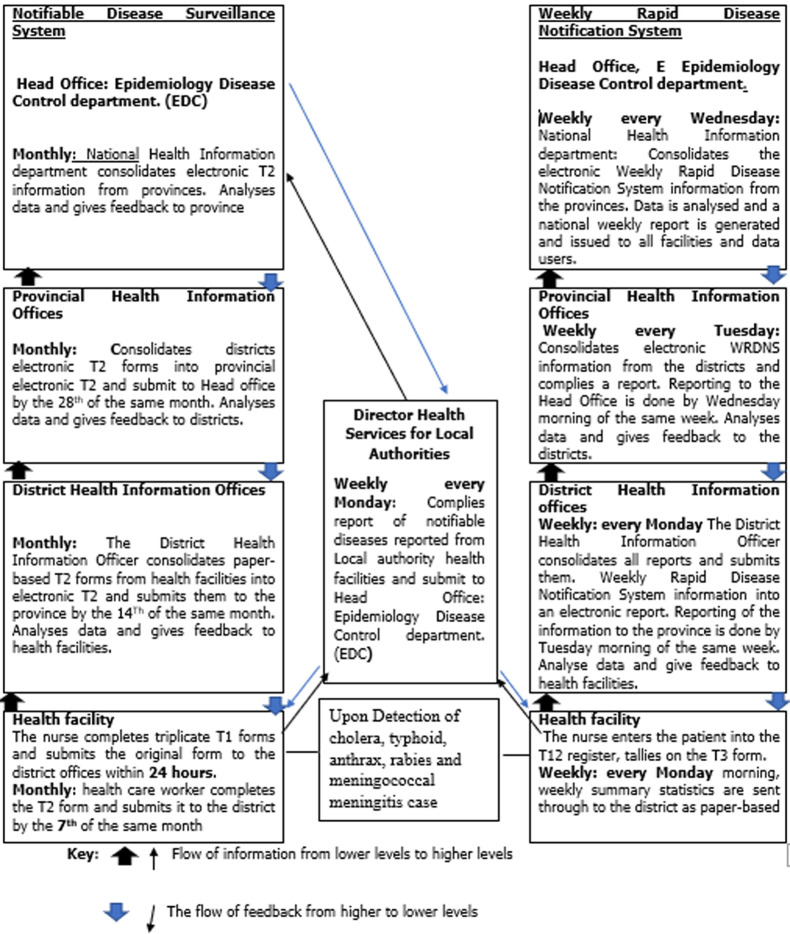
the information flow diagram in notifiable disease surveillance system and weekly rapid disease notification system adapted from Zimbabwe health information system strategy (2009-2014)

In the NDSS, any health care worker who encounters a suspected or confirmed case or the associated mortality cases of a notifiable disease notifies the district medical offices using the fastest means of communication. In Zimbabwe, according to the Public Health Act (15: 09) the notification form Tally 1 (T1) is used for notification of notifiable diseases [3]. Paper-based individual case reporting notification forms (T1) are completed in triplicate for the first five cases. One copy of the T1 form is kept in a file at the health facility, the second copy is sent to the district medical offices and the third copy is sent to the provincial medical offices. For more than five cases, a line list is maintained. At the end of the month, nurses at the primary health facilities complete and submit a consolidated paper-based monthly report for notifications Tally 2 (T2) form to the district medical offices. Tally 2 zero reporting should be done if no cases were notified. At the district level, the District Health Information officer (DHIO) enters all the data from a consolidated district paper-based T2 into the District Health Information System (DHIS) version 2.32.1 which is an electronic data repository that allows the integration of all reporting systems. District electronic T2 form is subsequently forwarded to the provincial medical offices by the 14^th^ day of the same month. The provincial medical offices consolidate all districts' electronic T2 forms into a provincial electronic T2 summary which is submitted to the Head Office by the 28^th^ day of the same month [6]. According to section 49 of the public health act, during an outbreak, local authorities should submit weekly reports to the Ministry of Health and Child Care Epidemiology and Disease Control department of cases and deaths from infectious diseases that would have been reported to them [3] (Figure 1). The weekly disease surveillance system was put in place to compliment the NDSS so that it reports some notifiable diseases weekly rather than waiting to be reported monthly through the NDSS thus, ensuring timely reporting and on-time public health action. Among the notifiable diseases in the NDSS, the following; cholera, typhoid, anthrax, rabies and meningococcal meningitis are reported weekly through the WRDNS in Zimbabwe [7].

A review of DHIS 2 notifiable diseases records in Chegutu District showed a discrepancy between the WRDNS, and the NDSS, with 106 cholera cases and 2 rabies cases having been reported through the WRDNS in 2018-2019. The electronic consolidated report for notifications T2 recorded 111 cholera cases as opposed to 106 cases reported in the electronic WRDNS. The sum of the weekly summaries of notifiable diseases in DHIS 2 at the end of the month should be the same as the respective figures in the electronic T2 of the NDSS monthly report for the same disease. Only one notification form Tally 1 (T1) form was completed in the Chegutu District instead of seven forms for the notified diseases (5 cholera and 2 rabies) in the 2018-19 period. Integrated Disease Surveillance and Response (IDSR) training were done to health care workers in the district as well as support and supervision of health care workers. Failure to detect outbreaks of emerging and re-emerging communicable diseases threaten the health and wellbeing of communities, and when uncontrolled, can lead to a global threat [8]. Data discrepancy and gaps in notification may reflect underreporting of cases in the NDSS, missed opportunities for monitoring and control of diseases. The district NDSS has never been evaluated. We evaluated the performance of the NDSS in the Chegutu District to come up with evidence-based recommendations to improve the surveillance system.

## Methods

**Study design:** we conducted a descriptive cross-sectional study using Centres for Disease Control and Prevention (CDC) Update Guidelines for Evaluating Public Health Surveillance Systems [1]. The CDC updated guidelines focus on demonstrating that a public health surveillance system provides information that enhances public health decisions and describes system usefulness and attributes, which included the following, simplicity, acceptability, flexibility, representative, data quality, completeness, stability, and timeliness [1].

**Study setting:** the study was conducted in Chegutu District, Mashonaland West Province in Zimbabwe. Chegutu is one of the seven administrative districts in Mashonaland West Province. It lies 107 kilometres southwest of Harare, along the Harare-Bulawayo Road. Chegutu District serves a projected population of 180,74 people [9]. The sources of livelihood in the district include indigenous companies, mining, commercial farming, and subsistence farming. The district recorded 111 cholera cases in 2018 and is considered a cholera hotspot in Zimbabwe [10]. The district has three hospitals and 31 clinics that participate in the NDSS.

**Study population:** our study populations were five doctors and 132 nurses in Chegutu District who participate in the NDDS. The District Medical Officer (DMO) and the District Nursing Officer (DNO), District Health Information Officer (DHIO) and the sister in Charge Community (SICC) were the key informants. Tally 1 notification forms were reviewed for completeness, timeliness, and data quality.

**Sample size determination:** the sample size was calculated using the Dobson formula [11]:


$$n=\frac{Z_{\alpha }^{2}(P)(1-P)}{d^{2}}$$

Using assumptions from a study by Mairosi *et al*. (2017) where health workers´ good knowledge of the NDSS was at 12% in Centenary District, Zimbabwe [12]. A 10% margin of error, a confidence interval (CI) of 90%, a non-response rate of 10%, a minimum sample size of 46 health workers was calculated.

**Sampling:** we purposively selected 14 high volume clinics and three hospitals in the district, and these represented 50% of the 34 health facilities in Chegutu. We used simple random sampling to select two nurses from clinics where all three nurses reported on duty on the day of the visit. In instances where only one nurse reported on duty on the day of data collection, we recruited him or her into the study. At the three hospitals in the district, we randomly selected five health care workers involved in the NDSS using random numbers generated by the RANDBETWEEN function in Microsoft Excel. We purposively selected the key informants who included the District Medical Officer, District Nursing Officer, District Health Information Officer and the sister in charge community. We reviewed the available T1 notification forms.

**Data collection methods and tools:** we used a pretested interviewer-administered questionnaire to collect data from the study participants. The questionnaire was used to collect information on the reasons for low notification of notifiable diseases, reasons for data discrepancy between WRDNS and NDSS, knowledge level on the NDSS system, to assess NDSS attributes which are; simplicity, flexibility, data quality, acceptability, representativeness, timeliness, stability and determine participants´ views on the usefulness of the surveillance system. We used a key informant interview guide to gather information on the performance of the NDSS. A checklist was used to check for the availability of resources of the NDSS to assess system stability.

### Definition and measurement of variables

**Data quality and completeness:** data quality reflects the completeness and validity of the data recorded in the public health surveillance system [1]. To assess data completeness, we reviewed the T1 notification forms to check if all 37 fields on the form were completely and correctly completed. Good data quality was defined as having all 37 sections completed and poor data quality as having any of the 37 sections incomplete.

**Flexibility:** the flexibility of a public health surveillance system is defined as the ability to adapt to changing information needs or operating conditions with little personnel, additional time, or allocated funds [1]. The flexibility of the T1 form was assessed by checking the ability of the T1 notification form to be added new information or new notifiable diseases that will have been identified.

**Acceptability:** acceptability is defined as the willingness of persons and organizations to participate in the public health surveillance system [1]. We assessed acceptability by asking the health care workers about their willingness to participate in the NDSS. We objectively checked for acceptability by checking for completeness of T1 notification forms and timeliness in reporting of T1 forms. Furthermore, we checked on health care workers´ attendance at NDSS meetings from a minute book.

**Simplicity:** simplicity refers to both structure and ease of operation of the public health surveillance system [1]. Simplicity was objectively assessed by asking and observing how easy it was to operate the system and fill in the T1 forms. From the IDSR training guidelines that are used to train health care workers on the NDSS, the T1 notification forms should take a maximum of ten minutes to complete when all the information needed is available.

**Timeliness:** timeliness reflects the speed between steps in a public health surveillance system [1]. To measure timeliness, we checked the dates T1 notification forms were completed, submitted to the district against the date of diagnosis of a notifiable disease. Timeliness was defined as the number of notifiable diseases that were notified to the district offices within 24 hours of diagnosis or reporting.

**Stability:** the stability of a public health surveillance system refers to the reliability and availability of the system [1]. Stability was measured by assessing the availability of resources, training and system performance. We checked the health care workers who were trained in IDSR which included the NDSS component.

**Flexibility:** a flexible public health surveillance system can adapt to changing information needs or operating conditions with little additional time, allocated funds or added personnel [1]. The flexibility of the NDSS was assessed objectively by asking and checking on the T1 notification forms to see if the system was adaptable to changing information.

**Usefulness:** the updated CDC guidelines describe a public health surveillance system to be useful if it contributes to the prevention and control of adverse health-related events [1]. Usefulness was measured by asking the participants their perceptions regarding the usefulness of the NDSS, public actions or decisions that were carried out or made based on the findings from NDSS data collected. We objectively checked for evidence of meeting minutes and public actions taken based on NDSS data.

**Health care worker knowledge:** a rating scale was used to assess health worker knowledge, where a rating of poor, fair and good was used [13]. We measured health worker knowledge by the use of eight questions on expected NDSS knowledge. Assuming that each correct response carries the same weight, the responses were graded as poor for 0-3 correct responses, fair 4-6 correct responses, and good for 7-8 correct responses.

**Data analysis:** Epi Info 7.2.4.0 software was used to generate frequencies, proportions, and means. Knowledge of health care workers on the NDSS was assessed using a 3-point rating scale which was rated as good, fair, and poor [13]. Qualitative data from key informants were grouped manually into themes and were analysed thematically, based on responses to specific questions.

**Ethics approval and consent to participate:** ethical approval for the study was obtained from the Mashonaland West Provincial Medical Directorate Institutional Review Board. Permission to carry out the study was obtained from Mashonaland West Provincial Medical Directorate, Chegutu District Medical Officer, the Health Studies Office. No participants´ names or addresses were used during the study. Collected data was kept in privacy. Confidentiality was maintained throughout the study. Written consent was obtained from the study participants. Since data collection was conducted during the COVID-19 pandemic era, social distancing, hand hygiene and the wearing of a face mask covering the nose and mouth were maintained.

## Results

Forty-six participants were recruited into the study and the response rate was 100%. The participants included registered general nurses, primary care nurses and medical doctors.

**Demographic characteristics of respondents in Chegutu District, 2020:** the majority 38 (83%) of study participants were females and 30 (65%) were registered, general nurses. The median years in service for participants was 10 (Q1 = 3, Q3 = 14). More than half 35 (76%) were employed by the government (Table 1).

**Table 1:** demographic characteristics of health workers in the notifiable disease surveillance system in Chegutu District, Zimbabwe, 2020

Variable	Frequency n=46	Percentage
**Sex**		
Female	38	83
Male	8	17
**Designation**		
Registered general nurses	30	65
Primary care nurse	14	31
Medical doctor	2	4
**Sector employed**		
Government	35	76
Rural council	7	15
Urban council	4	9
Years in service	Median 10 (Q1=3; Q3 =14)	

**Reasons for low reporting of notifiable diseases in Chegutu District , 2020:** the reasons for the poor performance of the NDSS reported by the participants were lack of training in NDSS 42 (91%), unavailability of IDSR guidelines 38 (83%), unavailability of T1 notification forms 8 (18%). All 17 facilities did not have IDSR guidelines. Only 3/17 of the health facilities had T1 notification forms which were adequate to notify the first five cases in case of an outbreak.

**Reasons for data discrepancies between NDSS and WRDNS:** the reasons for data discrepancy reported by participants were errors made by health care during counting and compiling reports 37 (80%) and typographical mistakes during data entry on WhatsApp 32 (70%).

**Health care worker knowledge assessment on the notifiable disease surveillance system in Chegutu District, 2020:** all 46 (100%) participants knew that notification of a notifiable disease was a statutory requirement. Most participants 36 (79%) knew that a notifiable disease should be notified within 24 hours of detection. Seventy-two per cent (33 participants) knew that T1 notification forms should be sent to the district medical offices. The majority 32 (70%) knew, that T1 notification forms were used for notification. Twenty-eight per cent (61%) knew the definition of a notifiable disease. Only six (13%) knew more than nine examples of notifiable diseases. Seventeen participants (37%) knew that T1 notification forms were completed in triplicate. Using a 3-point rating scale, the health care workers in the Chegutu District had fair knowledge 36 (78%) (Table 2).

**Table 2:** health care worker knowledge assessment of notifiable disease surveillance system Chegutu District, Zimbabwe, 2020

Variable	Category	Frequency n=46	Percentage
Know notification of a notifiable disease was a statutory requirement		46	100
Know a notifiable disease should be notified within 24 hours of detection		36	80
Know T1 notification forms should be sent to the district medical offices within 24hours		33	72
Know T1 notification form used for notification in the NDSS		32	70
Know the definition of a notifiable disease		28	61
Know T1 notification forms should reach district medical offices within 24 hours of case detection		28	61
Know 3 T1 notification forms should be completed during notification		17	37
Know examples of notifiable diseases	Mentioned <5	30	65
	Mentioned 5-8	10	22
	Mentioned 9 and above	6	13
Rating scale	Fair	36	78
	Good	6	13
	Poor	4	9

### Attributes of the NDSS

**Timeliness and data quality:** only one (1/17) health facility completed a T1 notification form from November 2020 to January 2021. Out of four notifiable diseases diagnosed, no TI notification forms were completed within 24 hours of a case reporting to the health facility. Only one out of the four diagnosed notifiable diseases had a T1 notification form which was completed correctly and adequately filled in all sections.

**Simplicity:** six health care workers who were timed and observed whilst completing T1 notification forms took six to eight minutes and had no difficulties in completing the forms. They described the process as easy. All 17 health facilities had more than five notifiable disease standard case definitions that they described to be easy to use.

**Representativeness:** all 17 health facilities in the district were participating in NDSS. However, the key informants highlighted that the five private health facilities in the district were not participating in NDSS.

**Acceptability:** the majority 37 (80%) of the participants felt it was their duty to complete the T1 notification forms and were willing to continue completing the forms. Assessed completed T1 notification form had no missing information rendering the system acceptable. The majority 37 (80%) of participants showed meeting minutes for attending weekly and monthly surveillance meetings on the NDSS.

**Stability:** one health care worker per facility was trained in IDSR which covers the NDSS component. There was no documented evidence of feedback by those who attended training in all 17 health facilities. All 17 health facilities did not have IDSR guidelines. Only 3/17 of the facilities had more than 15 T1 notification forms which were adequate to notify the first five cases in an outbreak.

**The usefulness of the NDSS:** most participants 45 (98%) of the participants reported that the NDSS was useful at their level. Twenty-two 22 (48%) of the participants reported that NDSS data was used to plan health education talks while 19 (41%) reported that NDSS data was used to plan community awareness campaigns. There was evidence of data use for COVID-19 contact tracing and resource mobilisation. Minutes for monthly surveillance meetings for the three months of 2020 were shown as evidence in 15/17 health facilities.

### Results from key informants

**Reasons for the underperformance of the NDSS:** we interviewed four key informants involved in the NDDS in the Chegutu District. The reasons for the underperformance of the NDSS highlighted by the key informants were lack of training in IDSR, negative attitude of health care workers who would not have attended workshops, and lack of IDSR guidelines.

**Reasons for data discrepancy:** the key informants highlighted that data discrepancy could be due to typographical errors when sending statistics through WhatsApp. The District Health Information Officer (DHIO) verifies with the health facilities source documents whenever an anomaly was identified.

## Discussion

We conducted a descriptive cross-sectional study to evaluate the NDSS in Chegutu District, determine reasons for data discrepancy and underperformance to make evidence-based recommendations to improve the system. The system was found to be useful, flexible and simple. However, was not on time, unstable, and not representative. Health workers demonstrated fair knowledge of NDSS. The reasons for the poor performance of the NDSS were lack of health worker knowledge and unavailability of T1 notification forms. There was no evidence of data discrepancy between the NDDS and WRDNS.

Our study revealed that health workers in the Chegutu District had fair knowledge regarding the NDSS. This could be attributed to the fact that health facilities in the district had at least one health care worker who was trained in IDSR which includes the NDSS component. However, there was no evidence of documented feedback from those who would have attended workshops. Lack of adequate knowledge by health workers could lead to failure to diagnose and report a notifiable disease thus, leading to delayed investigations and under-reporting. These findings are contrary to findings by Haakonde *et al*. (2018) in Zambia who noted that most health care workers had poor knowledge regarding the system as they lacked regular IDSR training and mentorship [14].

Our study revealed that the NDSS in the Chegutu District was not on time as the notification was done six days after case detection. This could be attributed to the fact that the district needed a laboratory confirmation of the notifiable disease before notification. Lack of IDSR guidelines could also contribute to the late notification. Failure to notify on time leads to delayed investigations, prevention and control measures. These findings are in line with findings by Randriamiarana *et al*. (2018) in Madagascar who reported that data quality was poor and not on time due to lack of guidelines and training of health care workers in IDSR [15]. The NDSS in Chegutu was found to be simple. Six health care workers who were timed and observed whilst completing T1 notification forms took six to eight minutes and had no difficulties in completing the forms. The availability of standard case definitions made the system simpler. The simplicity of the NDSS improves the performance of the system as health care workers notify cases without facing any challenges. These findings are consistent with findings by Adjei *et al*. (2017) in Ghana who reported that the system was simple due to the availability of case definition and the notification forms were simple to use [16].

The NDSS in Chegutu was not representative. Health facilities in the private sector were not participating in the NDSS, only public health facilities in the district were participating in NDSS. Failure of private facilities to report notifiable diseases might lead to missed opportunities for diseases prevention and control as well as under-reporting of cases. These findings are consistent with findings by Mandyata *et al*. (2017) in Zambia who reported that private health facilities were not participating in the NDSS despite efforts made for them to participate [17]. Similarly, Makinde and Odimegwu (2020) in Nigeria reported that private health facilities had poor compliance with disease surveillance resulting in missed opportunities for disease prevention and control [18]. The NDSS in the Chegutu District was flexible. A review of the T1 notification form shows a section on diagnosis which allows one to enter the diagnosis and comment on additional notifiable diseases that would have been declared. These findings are consistent with findings by Juru *et al*. (2015) in Zimbabwe who found the NDSS to be flexible as the T1 forms could accommodate emerging diseases [19].

The NDSS was acceptable as participants in the study acknowledged that there were the ones responsible for filling the T1 notification forms and were willing to continue participating in the NDSS. Acceptability of the system by health workers improves the performance of the system. These findings are consistent with findings by Juru *et al*. (2015) in Zimbabwe, who reported that NDSS was acceptable as health care workers were willing to participate in the system [19]. The NDSS in the Chegutu District was not stable. This could be attributed to the fact that most health facilities had no IDSR guidelines and no T1 notification forms. There was a lack of documented feedback from health care workers who attended IDSR workshops. Lack of resources poses a risk of health care workers not notifying cases leading to delayed prevention and control measures. Furthermore, the lack of feedback contributes to poor health care workers´ knowledge leading to underperformance of the NDSS. These findings are consistent with findings by Mairosi *et al*. (2017) in Zimbabwe who found that the NDSS was unstable due to a lack of resources such as T1 notification forms [12].

The NDSS in the Chegutu District was useful. Data was used for planning health education talks evidenced by health education plans displayed in the facilities and documented health education talks. Since there were no cases of other listed notifiable diseases that should be notified through the NDSS there was evidence of data use during the time of COVID-19 where data was used for contact tracing and resource mobilisation. These findings are consistent with findings by Benson *et al*. in South Africa who reported that the NDSS was perceived as useful as data was used for disease control and response [20].

Our study had some limitations. The study design was descriptive cross-sectional, and the participants´ answers were self-reported. Consequently, there may have been a possibility of interviewer bias and social desirability bias. However, where possible we gathered evidence to support responses from health facility records and we triangulated the responses with the key informants´ interviews. Purposive sampling of health facilities limits the generalisability of the findings to all health facilities in the district. The limitations do not limit interpretation of our findings and our NDSS evaluation in the Chegutu District provides useful information on the performance of the NDSS.

## Conclusion

In our NDSS evaluation, Chegutu District NDSS was useful, flexible, and simple. Healthcare workers demonstrated fair knowledge of NDSS. However, the system was not on time, unstable, and not representative and there was a need for improvement. All system attributes should function at an optimal level for the NDSS to meet its objectives. We recommended NDSS training and mentorship to all health workers in the district. Health care workers who would have attended workshops should document the feedback which will serve as a reference. The district to have ongoing duplication and distribution of T1 notification forms. Targeted NDSS training to be conducted for health facilities in the private sector.

**Public health actions:** based on the evidence from this study we discussed the importance of the NDSS with all interviewed health care workers. We presented the findings of our study in a District Health Executive meeting. Five hundred and ten TI forms and 34 electronic integrated diseases surveillance guidelines were distributed to 34 health facilities.

### What is known about this topic

- Zimbabwe has made strides in training health workers in integrated disease surveillance and response which encompass the notifiable disease surveillance system. Integrated disease surveillance is the backbone of disease prevention and control;
- Notifiable disease surveillance system data reporting from health facilities have been suboptimal in Zimbabwe.

### What this study adds

- The study adds information on continued poor reporting of notifiable disease which is a public health threat as early disease prevention and control measures might be delayed;
- Demonstrates that inadequate resources such as lack of notification forms Tally 1 (T1) and poor health worker knowledge affect the performance of surveillance systems.
